# The potential impact of clinical decision support on nonwaivered primary care clinicians’ prescribing of buprenorphine

**DOI:** 10.1093/haschl/qxad051

**Published:** 2023-10-11

**Authors:** Anthony W Olson, Jacob L Haapala, Stephanie A Hooker, Leif I Solberg, Caitlin M Borgert-Spaniol, Katrina M Romagnoli, Clayton I Allen, Lorraine D Tusing, Eric A Wright, Irina V Haller, Rebecca C Rossom

**Affiliations:** Research Division, Essentia Institute of Rural Health, Duluth, MN 55805, United States; Research Division, HealthPartners Institute, Minneapolis, MN 55425, United States; Research Division, HealthPartners Institute, Minneapolis, MN 55425, United States; Research Division, HealthPartners Institute, Minneapolis, MN 55425, United States; Research Division, HealthPartners Institute, Minneapolis, MN 55425, United States; Geisinger Research, Geisinger, Danville, PA 17822, United States; Research Division, Essentia Institute of Rural Health, Duluth, MN 55805, United States; Geisinger Research, Geisinger, Danville, PA 17822, United States; Geisinger Research, Geisinger, Danville, PA 17822, United States; Research Division, Essentia Institute of Rural Health, Duluth, MN 55805, United States; Research Division, HealthPartners Institute, Minneapolis, MN 55425, United States

**Keywords:** medications for opioid use disorder, primary care, X-waiver elimination, clinical decision support systems

## Abstract

Elimination of the X-waiver increased potential buprenorphine prescribers 13-fold, but growth in prescribing will likely be much lower. We explored self-assessments of nonwaivered primary care clinicians (PCCs) for factors affecting their likelihood to prescribe buprenorphine were the X-waiver eliminated (since realized January 2023) and the potential impacts of a clinical decision-support (CDS) tool for opioid use disorder (OUD). Cross-sectional survey data were obtained between January 2021 and March 2022 from 305 nonwaivered PCCs at 3 health systems. Factors explored were patient requests for buprenorphine, PCC access to an OUD-CDS, and PCC confidence and abilities for 5 OUD-care activities. Relationships were described using descriptive statistics and odds ratios. Only 26% of PCCs were more likely to prescribe buprenorphine upon patient request, whereas 63% were more likely to prescribe with the OUD-CDS. PCC confidence and abilities for some OUD-care activities were associated with increased prescribing likelihood from patient requests, but none were associated with the OUD-CDS. The OUD-CDS may increase buprenorphine prescribing for PCCs less likely to prescribe upon patient request. Future research is needed to develop interventions that increase PCC buprenorphine prescribing.

**Clinical trial registration:** ClinicalTrials.gov. Identifier: NCT04198428.

**Clinical trial name:** Clinical Decision Support for Opioid Use Disorders in Medical Settings (Compute 2.0)

## Introduction

There were an estimated 80 816 opioid-related overdose deaths in the United States in 2021, a continued worsening of the country's long-lasting epidemic.^1^ That same year, 9.2 million Americans aged 12 years or older misused opioids, while only 887 000 people received medication for opioid use disorder (MOUD), including people undergoing treatment for opioid misuse in previous years.^2^ In 2020, only 60% of US counties had at least 1 clinician with an X-waiver to prescribe the MOUD buprenorphine in a clinic office setting, which serves as the predominant entry point for health care in the United States.^3^ Notably, counties that lacked waivered clinicians were the same ones where access to MOUDs to address opioid use disorder (OUD) and overdose was needed most.^4^

**Figure 1.**
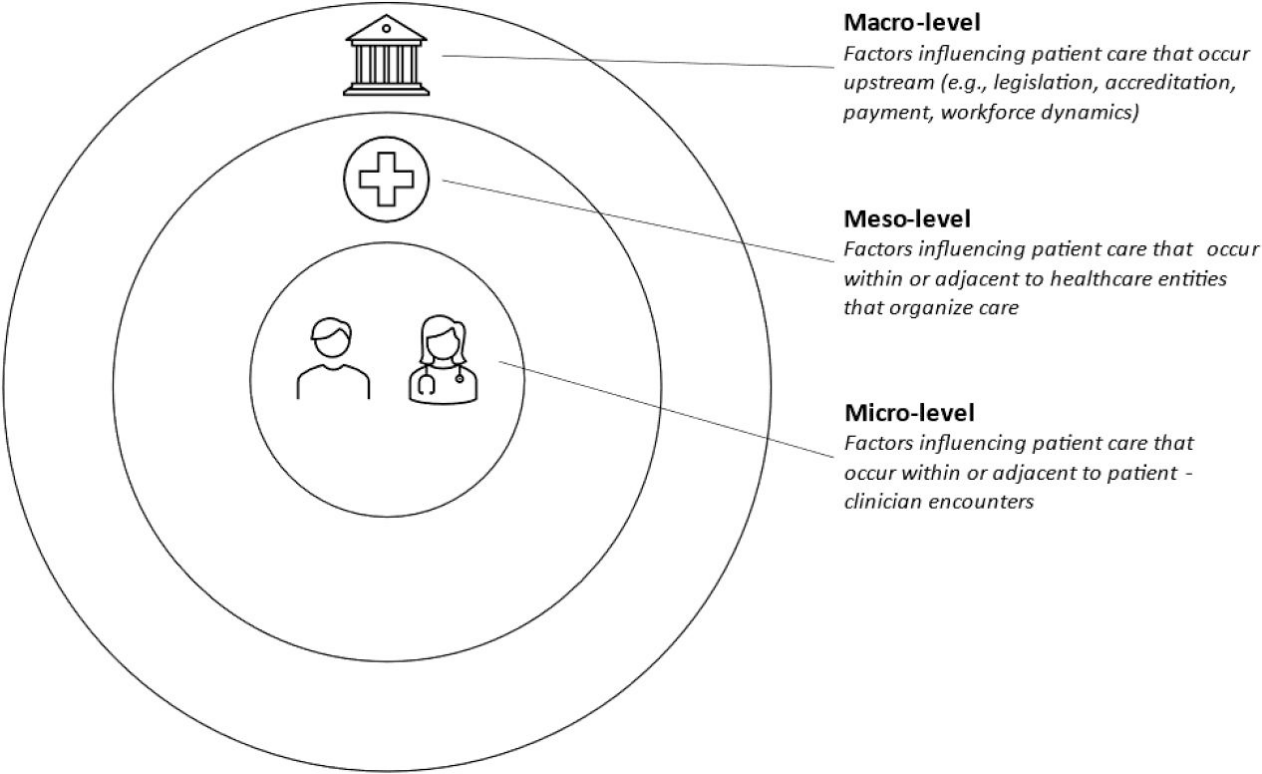
Three levels of factors influencing patient care. Source: Authors' depiction of definitions of macro-, meso-, and micro-levels presented in the figure are paraphrased from references 5 and 6.

An established framework conceptualizing where and how to use strategies to increase buprenorphine prescribing uses a concentric hierarchical structure of micro-, meso-, and macro-levels of interventional influence (see Figure 1).^5,6^ Micro-level factors influence patient care within or adjacent to patient–clinician encounters. Meso-level factors influence patient care within or adjacent to health care institutions that organize care. Macro-level factors occur upstream of patient care and include legislation and policy. While this 3-level framework avoids the complex overlapping and interdependence of these factors that occur in the real world, it parsimoniously highlights important context when examining intervention strategies.

The elimination of the X-waiver requirement in 2023 is the latest in a series of policy adjustments taking place at the macro-level, and it aims to increase buprenorphine prescribing by reducing regulatory barriers and social stigma surrounding OUD treatment.^7,8^ This regulatory change quickly and dramatically increased the potential pool of buprenorphine prescribers from 130 000 to 1.8 million.^9^ However, it remains to be seen if the elimination of the X-waiver will translate to change on the micro-level (ie, actual increased prescribing of buprenorphine among clinicians) since the clinicians recently added to the buprenorphine-prescribing pool did not actively seek a waiver ahead of the policy change. Even among the 55 000 US clinicians who had obtained an X-waiver after completing the time-intensive DATA 2000 training (8 hours for physicians; 24 hours for non-physicians) from April 2017 through January 2019,^10^ only about half (50.9%) wrote at least 1 buprenorphine prescription.^11^

Commonly cited barriers to MOUD prescribing often take place at the meso-level, including clinician lack of training and experience, the clinical complexity of buprenorphine induction with co-occurring disorders, and resource and capacity limitations, such as the inability to effectively identify at-risk populations or lack of time, institutional support, or support staff.^12-17^ Potential motivators include institutional support for treating OUD, OUD training or education, and patient demand for MOUD.^12,15^ An OUD-specific clinical decision-support (CDS) tool may be well suited for mitigating some of these identified barriers and facilitating increases in buprenorphine prescribing among primary care clinicians (PCCs) at scale. The Agency for Healthcare Research and Quality defines CDS as tools that provide “timely information, usually at the point of care, to help inform decisions about a patient's care…[that] help clinical teams by taking over some routine tasks, warning of potential problems, or providing suggestions for the clinical team and patient to consider.” Examples include order sets created for particular conditions or types of patients, recommendations, and databases that can provide information relevant to particular patients, reminders for preventive care, and alerts about potentially dangerous situations”^18^ While CDS tools can improve health care quality and patient outcomes by mitigating clinician barriers in providing care, better understanding of clinician perspectives about barriers to treating patients with OUD or whether an OUD-specific CDS could address them is needed.

To help address these barriers, our team developed a CDS tool (hereafter referred to as OUD-CDS) with features amenable to a streamlined care experience and high utilization by PCCs.^19^ The OUD-CDS was systematically designed in close collaboration with PCCs to support clinician screening, diagnosing, treating, and/or referring patients with or at high risk for OUD (eg, history of past overdoses, comorbidities, opioid prescriptions) while minimizing information overload, alert fatigue, and training and workflow adjustments to save the clinician time.^20,21^ All information and recommended actions of the OUD-CDS draw from patient-specific clinical data (eg, up to 2 years of pre-visit medications, diagnoses, labs, care encounters, opioid overdoses, hospitalizations, emergency department visits) combined with best-practice and safety monitoring guidelines.^22^ The OUD-CDS also uses algorithms to create educational handouts individualized to each patient that can be printed to prime patients to ask clinicians about managing OUD and to facilitate shared decision making.^23^ The intended result is a supportive institutional infrastructure that reduces clinical complexity and administrative burden for clinicians while also encouraging patients to recognize and request treatments that fit their needs.

The aim of this study was to explore factors related to PCC confidence and ability to appropriately care for their patients with OUD that might increase and improve the prescribing of buprenorphine, including the potential impact of a patient request for the medication and clinician access to a sophisticated OUD-CDS tool should the X-waiver be eliminated (since realized in January 2023).

## Data and methods

### Study setting and design

This study conducted a cross-sectional survey that was part of a 2-arm, clinic-randomized pragmatic trial to compare the effectiveness of PCC exposure to an OUD-CDS tool (ie, intervention) with usual care (ie, control). Both arms consisted of PCCs (ie, physicians, physician assistants, and nurse practitioners) caring for patients with or at risk of OUD in primary care settings.^22^ The overarching trial was performed at primary care clinics (*n* = 92) of 3 large health systems (Essentia Health, Geisinger, and HealthPartners) with primary care clinics in Minnesota, Wisconsin, North Dakota, and Pennsylvania. For this study, surveys were administered prior to each study site's intervention training modules before going live at each health system. The number of clinicians invited (windows for data collection) were 437 (January 5, 2021–March 23, 2021) at Health System A, 210 (June 22, 2021–November 8, 2021) at Health System B, and 308 (January 27, 2022–March 31, 2022) at Health System C. Intervention go-live dates were intentionally staggered for the overarching pragmatic trial so relevant lessons from 1 site could be passed on to others.^24^ Invitees who did not initially respond to invitations were emailed reminders weekly for up to 8 weeks.

### Data source

Survey data were collected via an electronic, self-administered survey using Qualtrics.XM software for survey management.^25^ Eligible participants were invited by research staff at their respective health systems to participate in the online survey via email. All PCCs in study-randomized clinics were eligible. For this study, only clinicians who indicated in the survey that they did not have an X-waiver were included for analysis.

### Variables

The study analyzed 8 survey items with 4- or 5-point Likert-type scales, with each item representing a unique variable. Three items measured PCC self-assessments of their confidence for screening, diagnosing, and referring patients with OUD. Three items measured PCC self-assessments of their abilities to provide motivational counseling, use treatment strategies for preventing overdoses, and refer to a specialist for patients with OUD. Two items measured willingness to prescribe buprenorphine should the X-waiver be eliminated (ie, patients with OUD requests buprenorphine and with an OUD-CDS tool available). Additional data were collected on respondent gender, race/ethnicity, practice degree, and days per week practicing in a clinic. The full-text survey is presented in Exhibit A1.

### Ethical and regulatory approval

The trial design and procedures were reviewed and approved by Institutional Review Boards (IRBs) at all 3 health systems. The IRBs at Health Systems B and C then ceded oversight of the study to the IRB at Health System A.

### Analysis

The Likert-type survey items related to the hypothetical likelihood of prescribing buprenorphine were described both at full scale and then dichotomized for further analysis. The 5 response options for prescribing likelihood if a patient with OUD requests buprenorphine were collapsed to “would” or “would not/unsure” and the 4 response options for agreement that having an OUD-CDS tool available would increase prescribing likelihood were collapsed to “agree” or “disagree” to ease interpretation of the practically meaningful findings (ie, PCCs stating they were more likely to prescribe buprenorphine with any degree of certainty). Chi-square tests were used to assess associations between the prescribing hypotheticals and demographic and practice-related characteristics. The responses for survey items related to confidence and ability to care for OUD patients were treated as continuous variables. For each prescribing hypothetical, we estimated odds ratios (ORs) for increased likelihood to prescribe buprenorphine (all “would” or “agree” responses) with higher levels of survey items related to confidence and ability to care for OUD patients (see Table A1). Adjusted ORs (aORs) were estimated by using generalized linear models with binomial error distribution and logit link functions, and all items related to confidence and ability were included as covariates. Two-sided tests were used and associations with *P* values <.05 were considered significant. Analyses were performed using SAS/STAT software, version 9.4 (SAS Institute, Cary, NC).

## Results

From 955 eligible PCCs invited to participate in the survey, there were 376 respondents (response rate = 39.4%). Of these, 314 clinicians did not have an X-waiver, and 305 of those provided usable responses. Nine of these 314 responses from 1 study site could not be used due to faulty skip logic when the survey was first launched, resulting in 305 usable responses for analysis.

Clinician demographic and practice-related variables are presented in Table 1. Across all 3 study sites, 64% of PCCs identified as female and 82% as White, 8% Asian, 2% Black/African American, 1% American Indian/Alaskan Native, less than 1% Native Hawaiian/Pacific Islander, 2% multiple/other races, and 2% of Hispanic ethnicity. The proportion of participants who reported practicing an average of 4 or more days in a clinic per week was 70%, with an additional 21% practicing an average of 3 days per week. Almost two-thirds of respondents were physicians (63%), with the remaining sample made up of nurse practitioners or physician assistants (37%). There were no significant differences between clinical demographic and practice-related groups for PCC willingness to prescribe buprenorphine upon patient request or with access to an OUD-CDS.

**Table 1.** Demographic and practice-related characteristics of nonwaivered primary care clinicians.

Characteristics	*n* (%)
Gender	
Male	105 (34)
Female	196 (64)
Third gender/nonbinary	1 (<1)
Prefer not to answer	3 (1)
Race/ethnicity	
American Indian/Alaska Native	2 (1)
Asian	25 (8)
Black/African-American	5 (2)
Hispanic/Latinx	7 (2)
Multiple/Other	6 (2)
Native Hawaiian/Pacific Islander	1 (<1)
Prefer not to answer	10 (3)
White	249 (82)
Average clinic practice days per week	
<1 day	4 (1)
1 day	8 (3)
2 days	16 (5)
3 days	64 (21)
4 days or more	213 (70)
Clinician type^a^	
Physician	187 (63)
Non-physician (nurse practitioner or physician assistant)	109 (37)

*n* = 305. Source: Authors' analysis of data collected in the pragmatic trial for reference 22.

Column percentages in each characteristic section may not sum to 100 due to rounding.

^a^Nine data points were not available for this variable.

Self-assessed factors potentially affecting the self-assessed likelihood of buprenorphine prescribing by nonwaivered clinicians are presented in Table 2. Only 26% of respondents reported that they “likely would” or “definitely would” prescribe buprenorphine if a patient with OUD requested it and a waiver were no longer required. Sixty-three percent of respondents strongly or somewhat agreed that access to an OUD-CDS tool would increase their likelihood of prescribing the medication to patients with OUD if an X-waiver were no longer required.

**Table 2.** Factors affecting self-assessed likelihood of buprenorphine prescribing among nonwaivered primary care clinicians prior to the elimination of the X-waiver.

	*n* (%)
Access to the OUD-CDS tool increases likelihood to prescribe buprenorphine	
Strongly agree	53 (17)
Somewhat agree	138 (45)
Somewhat disagree	64 (21)
Strongly disagree	50 (16)
Likelihood to prescribe buprenorphine if patient requested	
Definitely would	6 (2)
Likely would	72 (24)
Unsure	94 (31)
Likely would not	74 (24)
Definitely would not	59 (19)

Source: Authors' analysis of data collected in the pragmatic trial for reference 22.

Abbreviation: OUD-CDS, clinical decision-support tool for opioid use disorder.

Table 3 shows items rating confidence and abilities in diagnosing and treating patients with OUD for the overall sample as well as by self-assessed likelihood to prescribe buprenorphine (ie, all dichotomized “would” or “agree” responses) should the waiver no longer be required, when (1) a patient requests the medication or (2) the OUD-CDS tool was available. Overall, PCCs were more confident in their ability to know when to refer a patient with OUD to a specialist than in their ability to screen or diagnose their patients for OUD. The proportion of PCCs indicating low abilities in delivering brief motivational counseling, referring to an addiction care specialist, and using overdose-prevention treatment strategies were 30%, 30%, and 42%, respectively. Increased PCC confidence in diagnosing OUD and using overdose-prevention treatment strategies was positively associated with a higher likelihood of prescribing the medicine if requested by a patient with OUD (aOR [95% confidence interval (CI)]: 1.78 [1.08–2.98] and 1.71 [1.10–2.68], respectively). There was a marginal negative association between increased PCC confidence for when to refer to a specialist and a higher likelihood of prescribing the medicine if requested (aOR: 0.65; 95% CI: 0.41–1.00). Self-reported factors rating PCC confidence and ability to care for patients with OUD were not associated with the likelihood of prescribing the medicine if the OUD-CDS tool was available.

**Table 3.** Self-assessed factors affecting likelihood of buprenorphine prescribing among nonwaivered primary care clinicians by confidence and abilities in OUD diagnosis and treatment.

		Buprenorphine prescription if requested by patient with OUD				Buprenorphine prescription if OUD-CDS tool available			
Factor	Overall (%) (n = 305)	Would (%) (*n* = 78)	Would not/unsure (%) (*n* = 227)	*P*	aOR (95% CI)	Likely (%) (*n* = 191)	Not likely (%) (*n* = 114)	*P*	aOR (95% CI)
PCC confidence in screening for OUD									
Not at all confident	11	5	13	.37	0.78 (0.45-1.34)	11	11	.49	0.85 (0.52-1.37)
Somewhat confident	53	55	52			55	49		
Moderately confident	31	35	30			31	32		
Very confident	6	5	6			4	8		
PCC confidence in diagnosing OUD									
Not at all confident	17	9	20	.02	1.78 (1.08-2.98)	17	17	.82	0.95 (0.61-1.48)
Somewhat confident	46	45	46			45	47		
Moderately confident	31	37	29			34	27		
Very confident	6	9	5			4	10		
PCC confidence for when refer to specialist									
Not at all confident	7	10	5	.05	0.65 (0.41-1.00)	8	4	.79	0.95 (0.65-1.39)
Somewhat confident	30	21	34			28	33		
Moderately confident	49	60	45			51	45		
Very confident	15	9	17			13	18		
PCC ability to provide motivational counseling									
Low ability	3	1	3	.28	0.81 (0.53-1.20)	1	6	.28	1.20 (0.86-1.68)
Some ability	28	30	27			29	25		
Adequate ability	39	33	41			37	42		
Moderately high ability	28	33	26			31	23		
High ability	3	3	3			2	4		
PCC ability for using overdose-prevention treatment strategies									
Low ability	7	4	8	.01	1.71 (1.10-2.68)	5	11	.12	1.34 (0.92-1.96)
Some ability	36	30	39			37	35		
Adequate ability	43	45	42			42	44		
Moderately high ability	13	19	10			15	9		
High ability	1	3	1			1	2		
PCC ability to refer to specialist									
Low ability	4	4	4	.61	1.10 (0.76-1.59)	3	4	.25	0.83 (0.60-1.14)
Some ability	27	24	27			28	25		
Adequate ability	36	32	37			37	35		
Moderately high ability	27	31	25			26	27		
High ability	7	9	7			6	9		

*n* = 305. Generalized linear models included all PCC confidence and ability variables as covariates. Column percentages in each clinician response section may not sum to 100 due to rounding. Source: Author's analysis of data collected in the pragmatic trial for reference 22.

Abbreviations: aOR, adjusted odds ratio; CDS, clinical decision support tool; CI, confidence interval; OUD, opioid use disorder; PCC, primary care clinician.

**Figure 2.**
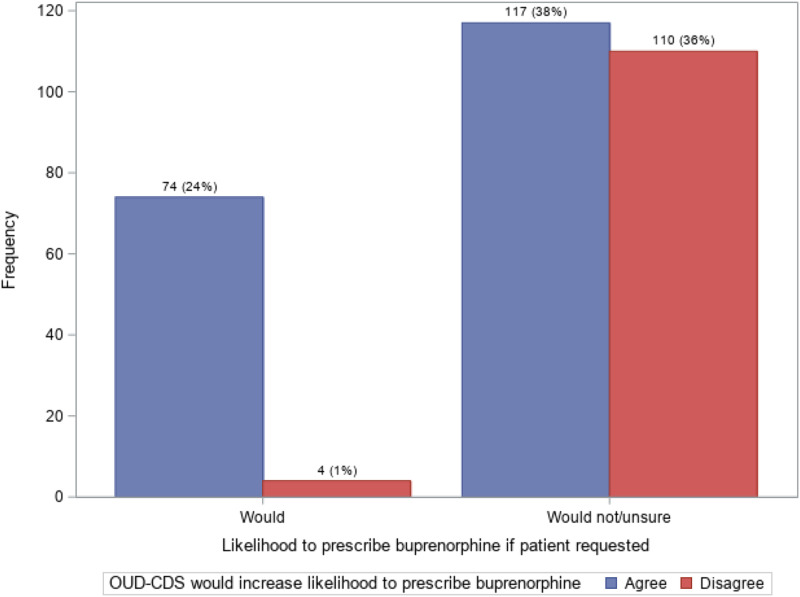
Frequency of buprenorphine-prescribing factor groups among nonwaivered primary care clinicians prior to the elimination of the X-waiver (*n* = 305). Source: Authors' analysis of data collected in the pragmatic trial for reference 22. Abbreviation: OUD-CDS, clinical decision-support tool for opioid use disorder.

Figure 2 crosses the 2 factors using the dichotomized responses (ie, all dichotomized “would” or “agree” responses) rating likelihood to prescribe buprenorphine (1) if requested by a patient with OUD and (2) availability of the OUD-CDS tool. Seventy-four clinicians (24%) responded that both a patient's request for buprenorphine for OUD and the availability of an OUD-CDS tool “likely would” or “definitely would” increase their likelihood of prescribing buprenorphine. The largest group of PCCs (38%) were those who “definitely would not,” “likely would not,” or were “unsure” if they would prescribe buprenorphine if requested by a patient with OUD but were likely to prescribe the medication if the OUD-CDS tool were available to them. Another 110 clinicians (36%) stated they “definitely would not,” “likely would not,” or were “unsure” if they would prescribe buprenorphine if requested by a patient with OUD and either strongly or somewhat disagreed that the OUD-CDS tool would make them more likely to prescribe the medication. The smallest group of PCCs (1%) were those who “definitely would” or “likely would” prescribe buprenorphine if requested by their patient with OUD but either strongly or somewhat disagreed that they were more likely to prescribe the medication if the OUD-CDS tool were available to them. Notably, among the 78 PCCs surveyed who reported they “definitely would” or “likely would” prescribe buprenorphine if requested by a patient with OUD (26% of total survey respondents), only 4 (5%) somewhat or strongly disagreed that the OUD-CDS tool being available to them would increase their likelihood to prescribe the medication. Conversely, among the 227 PCCs who reported they “definitely would not,” “likely would not,” or were “unsure” if they would prescribe buprenorphine if requested by their patient with OUD (74% of total survey respondents), there were 117 (52%) who agreed on some level that an OUD-CDS would increase their likelihood to prescribe the medication.

## Discussion

Although both the request for a buprenorphine prescription by a patient with OUD and the availability of the OUD-CDS tool may increase PCC prescribing of the medication post-elimination of the X-waiver, the availability of the OUD-CDS tool may have more influence on the likelihood of prescribing. Some PCCs’ self-assessed confidence and abilities for OUD care-related activities were independently related to the likelihood of PCCs prescribing buprenorphine if requested by a patient. However, self-assessed confidence and abilities were not associated with the likelihood of prescribing if the OUD-CDS tool were available. Also notable was that more than one-third (36%) of PCCs reported neither a patient request for buprenorphine nor access to the OUD-CDS tool would increase their likelihood to prescribe buprenorphine, even after the removal of the regulatory barriers that previously made it more difficult for them to do so.

The micro-, meso-, and macro-levels depicted in Figure 1 offer a useful lens for interpreting these findings.^5,6^ Micro-level factors in this study were represented by variables for PCC confidence and abilities to appropriately care for patients with OUD and the likelihood of prescribing buprenorphine for patients with OUD requesting the medication. Meso-level factors in this study were represented by the OUD-CDS's availability to PCCs. A macro-level factor in this study was represented by the hypothetical (and now actualized) elimination of the X-waiver. While this 3-level framework avoids the complex overlaps and interdependence of these factors in the real world, it parsimoniously highlights important context for each factor.

### Macro-level

The formal elimination of the X-waiver in January 2023 removed a macro-level barrier to buprenorphine access for patients with OUD by, among other things, enabling clinicians to prescribe the medication with a standard Drug Enforcement Administration (DEA) license. The policy change increased the number of potential prescribers and created conditions that may help reduce treatment hesitancy and stigma for both patients and clinicians.^26^ However, the suboptimal prescribing rate among waivered clinicians prior to the change illustrates the importance of factors taking place at the other levels of care.^11^ Thus, the study's finding that almost two-thirds (64%) of the nonwaivered PCCs reported being more likely to prescribe buprenorphine when accounting for factors acting at the micro- or meso-levels of care is promising news. Furthermore, other buprenorphine prescribing factors not examined in this study may hold influence among the 36% of nonwaivered PCCs who were not as willing to prescribe.

### Micro-level

Over one-quarter (26%) of nonwaivered PCCs in the sample said they were willing to prescribe buprenorphine if their patient with OUD requested the medication and the X-waiver were eliminated. The likelihood of clinicians reporting that they would prescribe buprenorphine was positively associated with incremental increases in their confidence to diagnose OUD and the ability to use overdose-prevention treatment strategies. Meanwhile, the self-assessed likelihood of prescribing buprenorphine when requested by a patient negatively related to incremental increases in their confidence in knowing when to refer to a specialist. There are several explanations for these relationships. For instance, clinicians with lower levels of confidence in diagnosing OUD would, of course, be less likely to prescribe treatment for the disease, especially with a medication like buprenorphine that requires additional training and approvals to obtain an X-waiver and prescribe. It is also unsurprising that, among a sample of nonwaivered PCCs without previous experience prescribing buprenorphine, clinicians felt more confident in knowing when to refer to an OUD specialist than in their ability to prescribe OUD treatment. However, it should be noted that there is a shortage of sub-specialist addiction medicine clinicians, which limits the effectiveness of relying on referrals. Finally, clinicians who perceive themselves as having insufficient abilities to use overdose-prevention treatment strategies, such as prescribing naloxone, would be unlikely to see themselves as having sufficient abilities to prescribe scheduled drugs for the same purpose. Less evident from the results are the causes behind lower levels of PCC confidence and ability. This may be attributable to inexperience, lack of education, disinterest, discomfort, stigma, or other perceived challenges involved in care for patients with OUD.^27-29^

The variables for PCC confidence in screening for OUD, ability to provide brief motivational counseling, and ability to refer to a specialist were not associated with the odds of PCCs stating they would prescribe buprenorphine when requested by a patient. These factors have a more nuanced relationship with buprenorphine prescribing than those described in the previous paragraph. For example, primary screening for diseases, including OUD, is conducted for patients without symptoms and is often completed by the patient prior to the encounter (eg, paper form given by the front desk or clinical assistant). Thus, PCCs may feel such verbal screenings are less needed in the encounter, and therefore less practiced regardless of their openness to prescribing for patients with OUD. Another interesting subtlety suggested from the results is that a PCC's self-assessed ability to promote health-behavior changes in their patients with OUD, such as willingness to take buprenorphine, was unrelated to an increased likelihood of clinicians to prescribe buprenorphine under the hypothetical elimination of the X-waiver. This may change over time as the elimination of the X-waiver gives PCCs more opportunities to prescribe buprenorphine in conjunction with brief motivational counseling, just like they would for patients with other chronic diseases.^30^ Finally, the ability of a clinician to refer a patient with OUD to a specialist may have had as much to do with an X-waivered clinician's proximity or availability as much as the PCC's technical knowledge and skills to refer. Based on 2020 DEA lists, almost one-third of all rural counties and more than half of remote counties in the United States did not have an X-waivered clinician practicing.^31^

### Meso-level

Almost two-thirds of nonwaivered PCCs (63%) in the sample stated they were more likely to prescribe buprenorphine if the OUD-CDS tool were available to them, whereas, in a separate question, only 26% reported they would prescribe buprenorphine if the patient requested the medicine. This suggests that the meso-level OUD-CDS tool may increase the likelihood of prescribing the medication beyond the micro-level patient request. The findings in Table 3 also show that the PCC factors behind the OUD-CDS tool's influence are unrelated to the PCC confidence and ability factors. Although future research is needed, this finding suggests a hypothesis that PCC prescribing of buprenorphine may be more influenced by meso-level factors within the health system's control than many micro-level factors outside of it (eg, patients with limited knowledge of MOUD, lack of a strong patient–provider relationship), with the notable exception of a patient's willingness to take medications like buprenorphine.

### Future research

Future research is needed to determine how well PCCs’ self-perceived likelihood to prescribe buprenorphine matches actual prescribing practices. Furthermore, additional investigation is needed to find any interactive and additive effect between a patient request for buprenorphine and the availability of the OUD-CDS tool, as these 2 factors were associated with increased likelihood of prescribing the medication. Finally, it is not clear what types of interventions would increase buprenorphine prescribing among PCCs who are similar to the 36% of this study sample reporting that neither a patient request nor access to the OUD-CDS tool would increase prescribing. Potential factors identified in the literature, which include issues related to utilization management requirements for buprenorphine (eg, prior authorizations), concerns about misuse or diversion of MOUD, resistance from practice partners, OUD-related care reimbursement rates, and mandates for OUD care from health system leadership, may be helpful in identifying key factors for this group of respondents, and stigma towards patients with OUD.^12-16,32,33^

### Limitations

There are several potential limitations created by methodological biases related to self-report measurements, survey instrument design, use of a hypothetical question, and sample generalizability. First, there may be poor reliability from self-report measures in surveys related to health care practice as well as potential for social desirability bias (ie, participants provide responses they perceive to be favorable to the investigators, peers, or their organization and society, especially if concerned about the anonymity or confidentially of their response). This suggests that more clinicians would provide responses indicating their willingness to adopt evidence-based guidelines for buprenorphine-prescribing practices after the elimination of the X-waiver than those who would actually follow through with doing so. Second, the 2 survey items measuring buprenorphine-prescribing factors in this study had inconsistent structures (4-point vs 5-point scales) and different phrasing (definitely vs strongly), which prevented stronger inferences. Additionally, survey items did not examine all potential factors potentially affecting buprenorphine prescribing among PCCs. Third, survey items were hypothetical in nature and were not an examination of what respondents did or did not do in the circumstances described. Finally, these results may only be generalizable to clinicians in large, integrated health systems like the ones included in this study. Clinicians who practice at smaller, independent clinics without easy access to similar resources may have different attitudes toward prescribing buprenorphine. Additionally, the response rate of 39% raises the possibility of volunteer bias for clinicians who were more open to prescribing buprenorphine than those who were not, given the stigma around the issue.^26^ Increases in PCC stress and time limitations attributable to the COVID-19 pandemic may have also contributed to a lower response rate.

## Conclusion

The recent elimination of the X-waiver through legislation aims to increase MOUD access by growing the potential pool of buprenorphine prescribers from 130 000 to 1.8 million amidst America's ever-worsening opioid epidemic.^9^ However, a reciprocal increase in buprenorphine prescribing seems unlikely given only about half of X-waivered clinicians wrote at least 1 buprenorphine prescription in the most recent year that data were available.^11^ Our study suggests that an OUD-CDS tool might further improve the likelihood of prescribing buprenorphine among PCCs, especially among those clinicians reporting they were not more likely to prescribe if the medication was requested by one of their patients with OUD. Future research is needed to investigate factors that may improve buprenorphine prescribing among PCCs who were less likely to do so if the medication was requested by a patient or an OUD-CDS tool was available to them.

## Supplementary Material

qxad051_Supplementary_Data
