# Supplementary material for: The potential impact of clinical decision support on nonwaivered primary care clinicians’ prescribing of buprenorphine
Source: Health Aff Sch. 2023 Oct 11;1(4):qxad051. doi: 10.1093/haschl/qxad051 (PMC10986287; doi:10.1093/haschl/qxad051)
Supplement: qxad051_Supplementary_Data [file qxad051_Supplementary_Data.zip › OWBaselineSurvSupMat-HlthAffSch_092123.doc]

**Appendix: Supplementary Material**

**Exhibit A1: Clinician Survey**

 Please answer the following questions to the best of your ability. Your answers help us understand the impact Opioid Wizard may have on clinician ability to diagnose and treat Opioid Use Disorder, and the usefulness of the tool.

With what gender do you identify?

- Male
- Female
- Transgender male
- Transgender female
- Non-binary
- Not listed
- Prefer not to answer

Do you consider yourself to be Hispanic/Latinx?

- No
- Yes
- Prefer not to answer

If "Yes", indicate the group that represents your Hispanic origin or ancestry (check all that apply):

- Puerto Rican
- Dominican (Republic)
- Mexican/Mexican American
- Chicano
- Cuban/Cuban American
- Central or South American
- Other Latin American
- Other Hispanic or Latinx
- Don't know
- Prefer not to answer

What race do you consider yourself to represent? (Check all that apply)

- American Indian or Alaska Native
- Asian
- Black or African American
- Native Hawaiian or Pacific Islander
- White
- Some other race (specify): ________________________________
- Don't know
- Prefer not to answer

On average, how many days a week do you see patients in clinic?

- 0
- 1
- 2
- 3
- 4 or more

Are you waivered to prescribe buprenorphine/Suboxone?

- No
- Yes

What is your buprenorphine waiver patient limit?

- 30
- 100
- 275
- Don't know

If your patient with OUD requested buprenorphine in the next year and a waiver were no longer required, would you prescribe buprenorphine?

- I definitely would not
- I likely would not
- Unsure
- I likely would
- I definitely would

Please answer the following questions about your knowledge and approach to management of patients with Opioid Use Disorder (OUD).

How confident do you feel screening your patients for OUD?

- Not at all confident
- Somewhat confident
- Moderately confident
- Very confident

How confident do you feel diagnosing patients with OUD?

- Not at all confident
- Somewhat confident
- Moderately confident
- Very confident

How confident do you feel treating patients with OUD with buprenorphine?

- Not at all confident
- Somewhat confident
- Moderately confident
- Very confident

How often do you treat patients with OUD with buprenorphine?

- Never
- Occasionally
- Sometimes
- Often
- Very often

How confident do you feel knowing when to refer your patients with OUD for treatment by addiction specialists?

- Not at all confident
- Somewhat confident
- Moderately confident
- Very confident

How often do you refer patients with OUD for treatment by addiction specialists?

- Never
- Occasionally
- Sometimes
- Often
- Very often

To what extent do you agree or disagree with the following statement: Having an EHR-integrated tool that coaches me on using buprenorphine for OUD would make me more likely to prescribe it, if a waiver were no longer required.

- Strongly disagree
- Somewhat disagree
- Somewhat agree
- Strongly agree

To what extent do you agree or disagree with the following statement: Having an EHR-integrated tool that coaches me on using buprenorphine for OUD would make me more likely to prescribe it.

- Strongly disagree
- Somewhat disagree
- Somewhat agree
- Strongly agree

Please rate your ability to effectively manage patients with the following treatment strategies:

|  | Low Ability | Some Ability; Could Use Improvement | Adequate Ability | Moderately High Ability | High Ability (On Par with Subspecialists) |
| --- | --- | --- | --- | --- | --- |
| Brief motivational counseling: |  |  |  |  |  |
| Overdose prevention: |  |  |  |  |  |
| Buprenorphine/Suboxone: |  |  |  |  |  |
| Referral for methadone: |  |  |  |  |  |
| Referral to specialty addiction care: |  |  |  |  |  |

**Table A2:** Coefficients and standard errors from regression models

|  | Buprenorphine prescription if  requested by patient with OUD | Buprenorphine prescription if  OUD-CDS tool available |
| --- | --- | --- |
| Intercept | -1.74 (0.63)** | 0.41 (0.56) |
| PCC Confidence in Screening for OUD | -0.25 (0.28) | -0.17 (0.25) |
| PCC Confidence in Diagnosing OUD | 0.58 (0.26)* | -0.05 (0.23) |
| PCC Confidence for when refer to specialist | -0.44 (0.23) | -0.05 (0.20) |
| PCC ability to provide motivational counseling | -0.22 (0.20) | 0.18 (0.17) |
| PCC ability for using overdose prevention treatment strategies | 0.53 (0.23)* | 0.29 (0.19) |
| PCC ability to refer to specialist | 0.09 (0.19) | -0.18 (0.16) |

**Note** N=305. * p < 0.05, ** p < 0.01, *** p < 0.001.

```
______________
 To access the Appendix, click on the Details tab of the article online.
```

^2^ To access the Appendix, click on the Details tab of the article online.
